# Time to go functional! Determining tumors’ DNA repair capacity *ex vivo*

**DOI:** 10.18632/oncotarget.26419

**Published:** 2018-12-07

**Authors:** Manuela Tumiati, Sakari Hietanen, Liisa Kauppi

**Affiliations:** Faculty of Medicine, University of Helsinki, Helsinki, Finland

**Keywords:** ovarian cancer, homologous recombination, triple-negative breast cancer, functional HRD, PAR Pi

The ability to better predict response to cancer therapy would substantially improve the lives of patients. For high-grade serous ovarian cancer (HGSOC) tumors, their DNA repair capacity - *via* the homologous recombination (HR) pathway - is the most important molecular determinant of sensitivity to platinum compounds, the DNA-damaging chemotherapy used in first-line treatment [1]. Namely, HR-deficient tumors are platinum sensitive.

To date, approaches to identify HGSOC patients with HR-deficient tumors have relied largely on DNA sequencing. They are based on scoring deleterious mutations in *BRCA1, BRCA2* and other known HR genes and/or identifying features of the genomic landscape, that is, so-called genomic scars and mutational signatures associated with HR deficiency. These methods carry limitations, however. First, HR deficiency caused by epigenetic alterations (e.g. *BRCA1* promoter methylation) is not detected by mutation screening. Second, mutations of uncertain significance may or may not confer HR deficiency. Third, genomic scars and mutational signatures associated with HR deficiency do not necessarily correspond to functional HR deficiency - HR-reversion mutations have been shown to restore HR functionality in tumor cells, while the "historical" genomic signature of HR deficiency is retained. Moreover, high-throughput sequencing remains comparatively expensive and necessitates heavy downstream bioinformatic analyses. Thus, it is not suited for determining tumors' HR capacity at the time when the sample is collected in surgery, nor in a routine clinical laboratory setting with limited resources.

**Figure 1:**
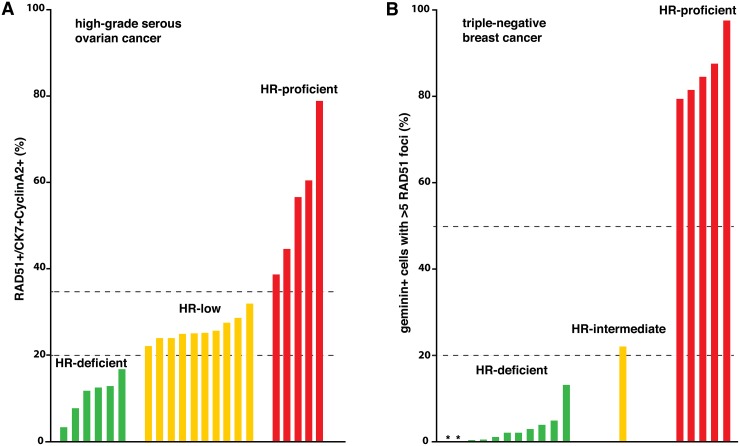
HR scores, as assessed by functional *ex vivo* testing, from 21 HGSOC patients (A, data from ref. 4) and 17 TNBC patients (B, data from ref. 5) Dashed lines indicate thresholds for the three HR capacity categories, as defined in the two studies. Asterisks in B mark the position of two TNBC samples with an HR score of zero [5].

Limitations of DNA sequence-based testing can be overcome by directly assessing whether the HR pathway is functional. To this end, detecting nuclear foci of RAD51, the key HR recombinase, has proven a powerful technique in classifying HR-deficient *versus* HR-proficient breast cancer samples [2, 3]. We set out to adapt this approach for HGSOC samples, and to validate its utility in predicting clinical platinum sensitivity. We obtained an HR score for each HGSOC patient sample by quantifying RAD51- mediated repair after ionizing radiation (IR) induced DNA damage in primary cell culture [4]. HGSOC samples clustered into three functional groups: HR-deficient, HR-low and HR-proficient (Figure 1A); genomic characteristics of HR deficiency were absent in one quarter of HR-deficient and HR-low samples. Low HR scores significantly correlated with platinum sensitivity, longer platinum-free interval and improved overall survival.

Shortly after our HGSOC study, Dik van Gent's group published a comprehensive analysis of HR capacity, as determined by RAD51 positivity *ex vivo* after IR-induced DNA damage, in 170 primary breast cancers [5]. The authors defined three sample groups, HR-deficient, HR-intermediate and HR-proficient (Figure 1B). Consistent with previous studies, HR gene mutations and genomic scars only partially associated with functional HR deficiency.

Given that the triple-negative breast cancer (TNBC) subtype is molecularly similar to HGSOC [6], it is now possible to compare the scope and frequency of HR deficiency in HGSOC *versus* TNBC (Figure 1). Interestingly, HR status in TNBC appears bimodal: all except one sample can be classified as clearly HR-deficient or HR-proficient (Figure 1B). In contrast, HR scores of HGSOC samples form a continuum, and the most common category in our cohort was low-proficiency HR (Figure 1A). This presumably reflects higher heterogeneity in HGSOC tumors compared to TNBC, and carries the implication of more varied long-term responses to DNA-damaging chemotherapy in HGSOC.

Another facet of HGSOC tumor heterogeneity uncovered in our study was that different anatomical sites within a single patient displayed a wide range of HR capacity. Of the five locations sampled at the time of surgery, one tumor (omentum) was HR-deficient, three tumors (left ovary, ascites and peritoneum) were HR-low and one (right ovary) was HR-proficient, resulting in an average of HR-low classification for this patient [4]. This so far anecdotal example highlights the tumor complexity that exists in therapy-naïve HGSOC patients. Such complexity will likely contribute to relapse events, even if primary chemotherapy is successful. It should be taken into account when sampling patient tumors for functional HR testing; a single anatomical site may not give a reliable estimate of HR capacity.

With the emergence of a new class of drugs, poly(ADP-ribose) polymerase (PARP) inhibitors in the clinic, functional read-outs of HR deficiency in breast and ovarian cancer are more important than ever before. For the repair of chemotherapy-induced DNA damage, HR-deficient tumor cells rely heavily on alternative pathways that are dependent on PARP activity. Blocking these pathways using PARP inhibitors (PARPi) generates a synergistic cell-killing effect known as synthetic lethality [7].

Patients with HR-deficient (that is, platinum-sensitive) tumors benefit significantly from PARPi maintenance therapy [8-10] but so far, methods to reliably identify this cohort have been lacking [8]. *Ex vivo* functional HR testing in two poor-prognosis cancer types, HGSOC and TNBC, has been now shown to be feasible [4, 5]. Determining the molecular phenotype (rather than genotype) of platinum-sensitivity will be central in comprehensively identifying those cancer patients who can benefit from PARPi therapy.
